# Impact of atmospheric condition on aerosol optical properties in urban and rural environment

**DOI:** 10.1016/j.apr.2025.102884

**Published:** 2026-05

**Authors:** S. Kłapiński, K.M. Markowicz, I.S. Stachlewska

**Affiliations:** University of Warsaw, Faculty of Physics, Institute of Geophysics, Pasteura 5, 02093, Warsaw, Poland

**Keywords:** Aerosol, Aerosol optical depth, Aerosol scattering coefficient, Black carbon, Angstrom exponent

## Abstract

This study investigates how aerosol optical properties derived from in situ and remote sensing observations differ between an urban site (Warsaw) and a background site (Strzyżów), under varying meteorological conditions. The analysis focuses on the role of air mass advection and vertical mixing, aiming to distinguish between air mass transformation effects and urban pollution impact. For this purpose, data from nephelometers, aethalometers, radiometers, and sun photometers supported by information on air mass trajectories are used. In spite of the fact that mean values of aerosol scattering coefficient (ASC) and equivalent black carbon (eBC) concentration are significantly different in urban and background sites, during northern flow but also during daytime convective conditions, the difference is relatively small. The highest difference has been found during southern flow or during nighttime conditions. Based on differences in aerosol optical properties during air mass transport between both sites, the air mass transformation effect and the urban effect were estimated. During transport of air mass between both sites the ASC, aerosol optical depth, and eBC increase by 39 %, 18 %, and 32 % in relative to the background site. Enhancement of these parameters due to urban effect was estimated as 32 %, 15 %, and 43 %. Comparison of the aerosol properties at both sites during the warm season shows negligible differences in ASC during clear-sky daytime and relatively small differences in eBC, as a result of intensive vertical mixing. ASC and eBC show significant variability with planetary boundary layer height (PBLH). The ASC can be reduced even up to 5.5 times when PBLH increases from 50 to 650 m at 12 UTC. During the warm season sensitivity of ASC and eBC to PBLH variability is significantly smaller than in the cold season.

## Highlights

• Daytime convection during the warm season improves urban air quality to level of background conditions.
• Both surface and columnar aerosol optical properties at urban and background site are statistically correlated.
• Air quality in Warsaw is more likely to be controlled by weather conditions than by temporal emission variability.

## 1 Introduction

Air quality is a critical factor influencing both environmental processes (climate, weather) and human health, especially in urban areas where pollution levels are typically elevated due to industrial activities, transportation, and dense populations (Chang et al., 2023; Kinney, 2008; Rafaj et al., 2021). Cities often experience higher concentrations of particulate matter (PM), for many chemical species such as nitrogen oxides, sulfur dioxide, and volatile organic compounds, which contribute to poor air quality and adverse health effects (Karagulian et al., 2015;Marlier et al., 2016; Kuntic et al., 2023). In contrast, rural or background sites, located far from major anthropogenic sources of pollution, generally record lower levels, providing a reference for assessing the impact of human activities and long-range transport (Putaud et al., 2010). Comparing urban and background air quality is essential to quantify the contributions of local emissions, regional transport, and natural sources such as dust or sea salt.

Recent advances in urban PM_2.5_ monitoring increasingly rely on multi-source observational frameworks that integrate ground-based measurements with satellite-derived aerosol products (Porcheddu et al., 2024; Li et al., 2023). Satellite AOD retrievals have been widely used to estimate near-surface PM_2.5_, although their accuracy strongly depends on atmospheric conditions (e.g., humidity, vertical aerosol profiles) and surface characteristics (e.g., surface reflectance) (Chen et al., 2024). To address these limitations, chemical-transport models are often incorporated to provide vertical information and improve spatial coverage in regions with sparse observations (Friberg et al., 2018). Machine-learning techniques have further enhanced these hybrid approaches by enabling nonlinear data fusion and bias correction in urban environments (Lolli, 2025; Porcheddu et al., 2025). Studies combining these methods have demonstrated substantial improvements in the accuracy and spatial resolution of PM_2.5_ estimates, particularly in densely populated areas where pollution gradients are strong (Saiohai et al., 2023; Li et al., 2023).

Many physical and chemical factors, such as emission, deposition, photochemical processes, as well as meteorological conditions, control the air quality in a given area in temperate latitudes (Li et al., 2024). In particular, the instantaneous pattern of high and low-pressure systems influences air circulation and horizontal and vertical mixing processes (Grundstrom et al., 2015; Ormanova et al., 2020 a; Lu et al., 2024). Advection is crucial for pollution levels, while the lifetime of aerosol in the atmosphere is a few days (Myhre et al., 2013). Also, convection leads to the distribution of pollutants in the air (Di Bernardino et al., 2021). In the well-mixed planetary boundary layer (PBL) the vertical concentration gradient is often small, however, due to the hygroscopic growth can significantly increase ASC with altitude (Pérez-Ramírez et al., 2021).

Air quality is commonly described by particulate matter mass concentration (PM) for particles smaller than 2.5 μm (PM_2.5_) and smaller than 10 μm (PM_10_). However, aerosol optical properties such as ASC measured by nephelometers at science research sites provided additional insights. The relationship between ASC and PM mass concentration, expressed as called mass scattering efficiency (MSE), varies with aerosol chemical composition i.e. the content of ammonium sulfate, ammonium nitrate, particulate organic material, sea salt, coarse mass, and particle size (Titos et al., 2012). A review of measurement results from (Hand and Malm, 2007; Shao et al., 2023; Chow et al., 2002) showed significant variation in MSEs, which range from 1.65 to 7.6 m^2^/g for PM_2.5_ particles and for large particles from 1.03 m^2^/g (PM_1_) to 4.5 m^2^/g (PM_10_). Another study approximated the relationship between the ASC and the corresponding PMs by a linear relationship, obtaining correlation coefficients of 0.83 for PM_1_ 0.81 for PM_2.5,_ and 0.65 for PM_10_ (Markowicz and Chiliński, 2020). The PM_2.5_ and PM_10_ changes depending on height of the boundary layer are expected, and can be significantly different at night and during daytime (Wang et al., 2019, Wang et al., 2023; Suchánková et al., 2024).

The motivation for this study comes from preliminary results and quick looks published at aerosol research network Poland-AOD web page (www.polandaod.pl, *last access November 22, 2025*), which shows that is-situ aerosol single-scattering optical properties at urban site in center of Warsaw can approach values observed at mountain background site in Strzyżów, particularly during spring and summer season. This study examines aerosol optical properties in both urban and background environments, analyzes the variability in pollutant levels, and identifies key meteorological factors influencing these differences. We hypothesize that under specific meteorological conditions—particularly during convective daytime periods and northerly air mass advection—the aerosol single-scattering optical properties at the urban site become similar to those at the background site due to enhanced vertical mixing and regional-scale air mass homogenization. The main goal of this research is to determine air masses and convective conditions impact on the difference of aerosol single-scattering properties at both sites. Based on that estimation of the transformation of air mass over Poland and the contribution of the megacity on the pollution level. The observational setup, including equipment and data processing, is described in Section 2. The following paragraph is focused on the mean aerosol optical properties at urban and background sites. In Section 4 the variability of aerosol properties due to air mass advection is discussed. The impact of convection mixing on the difference in aerosol single-scattering properties at both sites is presented in Section 5. The conclusions are found in Section 6.

## 2 Methodology

### 2.1 Research sites

**Fig. 1:**
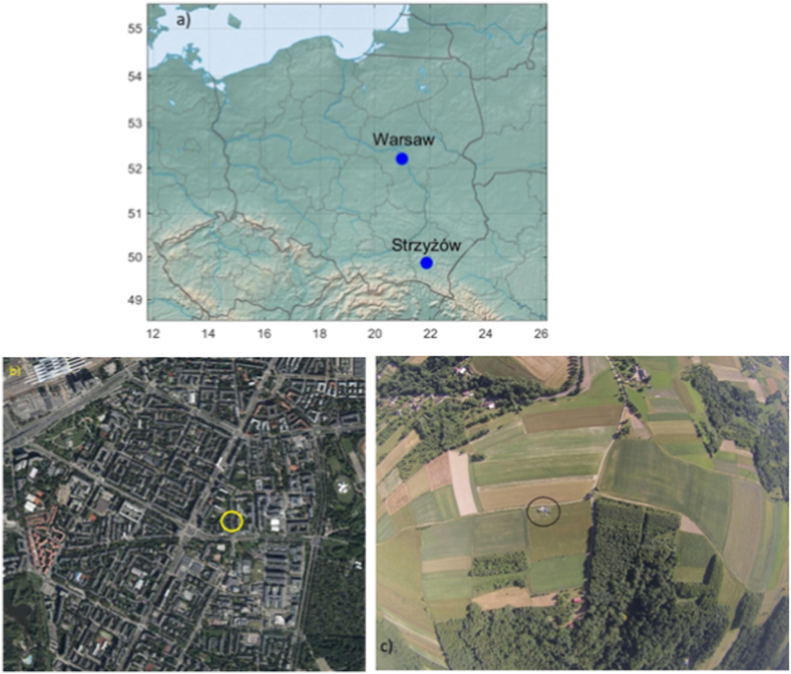
(a) Localization of the Warsaw and Strzyżów sites. Surroundings of the measurement site in (b) Warsaw (www.geoportal.gov.pl) and in (c) Strzyżów.

In this study, data from two research sites in Poland (Fig. 1a) is used. The first site in Warsaw (1.8 million inhabitants) is located on the roof of the Faculty of Physics building at the University of Warsaw (52°13′ N, 20°59′ E, 115 m above sea level). The site's surroundings (Fig. 1b) are typically urban: dense buildings, the presence of a four-lane road in the immediate vicinity of the site, and proximity to the city center - the distance to the Palace of Culture and Science is 2.8 km in a straight line. In the city and its surroundings, there are heating plants, power plants, and factories emitting air pollutants. The inlet for aerosol sampling is located about 20 m above the ground.

In the Strzyżów municipality (20,000 inhabitants), there is a second research site (49°53′ N, 21°52′ S, 444 m above sea level). The municipality is urban-rural, and the site itself is located away from the city center, in an undeveloped area (Fig. 1b). The site's surroundings consist of agricultural land, forests, and single buildings. Potential sources of pollution are emissions from household heating. However, in the immediate vicinity of the site (a few hundred meters), there are only single buildings. The distance from the center of Strzyżów is 5 km, and from Rzeszów, 25 km.

Both sites provide data to the national aerosol research network Poland-AOD and are part of the European research infrastructure for aerosol, clouds, and trace-gases observations ACTRIS. In this study, we analyzed in-situ data collected from May 2022 to April 2025, as well as remote data (sun photometer) the period from 2018 to 2025.

### 2.2 Equipment

The Cimel sun photometer is used to measure AOD and AE from direct spectral solar flux. Both instruments as part (Warsaw since 2018, Strzyżów since 2013) of the AErosol RObotic NETwork (AERONET; Holben et al., 1998) operated by the National Aeronautics and Space Administration (NASA) and PHOTONS (PHOtométrie pour le Traitement Opérationnel de Normalisation Satellitaire; University of Lille, CNES, and CNRS-INSU). At the Warsaw site the CE318-T Cimel is installed to perform sun, sky and lunar light measurements (Barreto et al., 2016). At the Strzyżów site the CE318-DP dual polarization (Li et al., 2009) is operated. Both sun photometers are equipped with 9 wavelengths (340, 380, 440, 500, 675, 870, 936, 1020, and 1640 nm), where AOD is calculated at 8 wavelength and total water vapour content or precipitable water (PW) at 936 nm. Ångstrom exponent (AE) is computed from AOD measured at 440 and 870 nm. Both instruments are calibrated once a year at University of Lille.

The aerosol scattering properties, such as ASC, and scattering Ångstrom exponent (SAE) were measured by a three-wavelength (450, 525, 635 nm) polar nephelometer (Aurora 4000, Ecotech, Melbourne, Australia; (Chamberlain-Ward and Sharp, 2011) Chamberlain-Ward and Sharp, 2011) at both sites. The measurement system is equipped with a movable cover that allows movement in 17 different configurations from 10 to 90°, thus allowing the measurement of the phase function shape. In this study, only the total ASC (from 10°) is used. Due to the relatively small spectral range of Aurora 4000, the SAE is calculated from a log-log fit of ASC to all wavelengths. Approximately every 3–6 months, the instrument must undergo a full calibration procedure. Full calibration involves adjusting both the range points and the zeros on the calibration curve. For this purpose, a gas (in this case, CO_2_) with known optical properties is used. Full calibration should be performed when the zero point changes by ± 2 Mm^−1^ or when the range points change by ± 1 %. The zero check is performed every day at 00 UTC and is subtracted from the raw Aurora 4000 signal.

The AE-33 (Drinovec et al., 2015) and AE-31 aethalometer, produced by Aerosol Magee Scientific are used to measure the equivalent of black carbon (eBC) and aerosol absorption coefficient (AAC), respectively in Warsaw and Strzyżów. Both devices measure the time variability of light transmittance (attenuation) through the Pallflex Q260F (AE-31) and TFE-coated glass M8020 (AE-33) filter with deposit aerosol on it. Based on the time variability of the attenuation coefficient, the eBC is estimated at seven wavelengths (370, 470, 520, 590, 660, 880, and 950 nm). The AE-33 was configured to measure the attenuation at 1 min resolution and airflow speed of 5.0 l/min, while AE-31 with 5 min resolution and 4.0 l/min flow speed. The main difference between the two devices is as follows. In the AE-33, two measurements are obtained simultaneously from two sample spots with different rates of accumulation of the sample, while in AE-31 is a single spot. The double spot technique in AE-33 allow to removal of nonlinearities and provide the compensated particle light absorption and eBC mass concentration. Comparison of AE-33 with AE-31 by Wu et al. (2024) shows AAC slopes ranging from 0.87 to 1.04 (for 7 wavelengths) and eBC slope of 1.9 (at 880 nm). In case of absorption Ångström exponent (AAE) defined for 370 and 950 nm, the agreement between both devices is much worse (Pearson correlation coefficient of 0.74) in comparison to eBC. Typical uncertainties in the ASC measured by aethalometers are on the order of 5–15 %, while uncertainty introduced by the assumed mass absorption cross-section (MAC) for converting ABS to eBC commonly adds ≈20–40 % uncertainty. Taken together, these contributions imply a realistic combined uncertainty on eBC of roughly 25–50 % under field conditions (Yus-Díez et al., 2021).

Both aerosol in-situ instruments (the nephelometer and the aethalometer) are connected to a common inlet (without particle size limitation). The aerosol inlet is mounted at a height of 2 m above the rooftop in Warsaw and 1 m above the container in Strzyżów to minimize local flow disturbances. In both cases, the sampled air is not dried before measurement. For the aethalometer, only rapid changes in relative humidity may affect the measurements, whereas for the Aurora 4000 nephelometer, high relative humidity can increase the scattering coefficient. However, only 5.3 % of the data in Warsaw and 9.7 % in Strzyżów were recorded when the relative humidity inside the Aurora 4000 exceeded 50 %. To remove possible hygroscopic effects, we excluded nephelometer data when relative humidity exceeded 50 %. To measure the solar radiation flux, the CMP21 (Strzyżów) and CMP22 (Warsaw) pyranometers were used. The spectral range of CMP21 is 285–2800 nm, while CMP22 is 200–3600 nm. Both instruments operate over a full 180° range. These radiometers also allow for the determination of clear-sky (cloudless) conditions. Theoretically, the CMP21 can recognize such conditions with an accuracy of up to 0.1 %, and the CMP22 up to 0.04 %. Both instruments are calibrated with the Poland-AOD network (Markowicz et al., 2022).

The sensible heat flux at the Warsaw site was measured by the Young 81000 sonic anemometer. It provides wind speed accuracy of ±0.05 m/s or ±1 % of reading and wind direction accuracy of ±2°, enabling reliable characterization of both mean flow and turbulent fluctuations. The instrument records data at a frequency of 32 Hz, making it well-suited for the eddy covariance method to estimate the heat flux.

### 2.3 Models

The Hybrid Single-Particle Lagrangian Integrated Trajectory model (HYSPLIT) is a numerical model used to calculate the backward trajectories of air particles and pollutants, as well as to simulate their dispersion, transport, deposition, and chemical reactions occurring in the atmosphere (Stein et al., 2015). To calculate backward trajectories, we used the Global Data Assimilation System (GDAS) with a resolution of 1° × 1°. The backward trajectories were calculated for a height of 500 and 1500 m at 00 and 12 UTC to determine the direction of air mass advection and also for the estimation of PBL height. The 2-day trajectory was used to select the direction of pollution transport. The ERA5 reanalysis dataset was used to derive hourly estimates of PBLH (Hersbach et al., 2020). ERA5 is produced by the European Centre for Medium-Range Weather Forecasts (ECMWF) on behalf of the Copernicus Climate Change Service (C3S) and is available from the Copernicus Climate Data Store.

### 2.4 Data processing

Observation performed at both sites is evaluated in frame of Poland-AOD network procedures (Markowicz et al., 2022) and by AERONET ver. 3 algorithm (Giles et al., 2019). Aurora 4000 data processing included the following corrections: zero calibration (Rayleigh scattering), non-Lambertian illumination, and the angular truncation error according to Müller et al. (2011) methodology modified by Markowicz et al. (2022). In case of the AE-33 eBC mass concentration is obtained from (Yus-Díez et al., 2021)

(1) $$eBC=\frac{A\Delta {ATN}_{1}}{Q_{1}\left(1-\zeta \right)\sigma_{ATN}C\left(1-k\Delta {ATN}_{1}\right)\Delta t}$$

where A is the filter surface area loaded with the sample, Q_1_ the volumetric flow of spot 1, *ζ* the lateral airflow leakage, $\sigma_{ATN}$ the MAC, *k* the loading factor parameter, C the multiple-scattering parameter *C*, and ΔATN_1_ the difference in the attenuation coefficient of the filter tape loaded with the sample of spot 1 during the measurement timestamp Δ*t*. For the AE-31 the eBC is estimated as following

(2) $$eBC=\frac{A\Delta ATN}{Q\sigma_{ATN}k\Delta t}$$

The manufacturing setting of AE-33 for the multiple-scattering parameter is 1.39, while the k loading factor parameter is computed based on two-spot measurements (Drinovec et al., 2015). For both AE-31 and AE-33, the MAC was set at 16.6 m^2^/g. In case of the AE-31 the k loading factor parameter is estimated based on the Collaud Coen et al. (2010) methodology. Such corrections are based on measured ASC and SAE, which are used to define the optical properties of all aerosols deposited on the filter for both the filter loading and scattering corrections. Due to the fact that previous research reports different multiple-scattering parameter *C* (e.g, Yus-Díez et al., 2021) we compared both instruments in Warsaw (July 2024). Fig. S1a in the Supplement shows a comparison of 1-h eBC from AE-33 and AE-31. We found a very high correlation coefficient (0.98) between both instruments; however, eBC at 880 nm for AE-33 is significantly higher than for AE-31. The slope is 1.189 ± 0.027 and offset is negligible (−4.6 ± 16.1 ng/m^3^). Based on that, we estimated the new multiple-scattering parameter of 1.65 (at 880 nm). After correction of the AE-33 data, the root mean square error is 5.5 ng/m^3^ for eBC. Our slope is close to the Wu et al. (2024) results. They report the AE33/AE31 slope eBC of 1.2 but this value is location dependent. In the case of AAC, the slope between our AE33 and AE31 is 2.361 ± 0.051 and almost zero offset (0.014 ± 0.216 Mm^−1^). For the AAE, the slope is 0.66 ± 0.065, the offset is 0.23 ± 0.09, and the correlation coefficient is 0.75. Finally, the single scattering albedo (SSA) at 525 nm is calculated from ASC and AAC.

The optical properties in Warsaw and Strzyżów were compared when the air mass trajectory passed in the vicinity both locations. For this purpose, we assumed a 100 km radius. For the southern flow, we selected the HYSPLIT back trajectories ending in Warsaw at 500 m a.g.l. that crossed within a 100 km radius around the Strzyżów site. This type of circulation is associated with the southerly flow (mostly a tropical air mass). Similarly, for the northern flow, we selected the back trajectories ending in Strzyżów when they crossed within the same radius around Warsaw. This case mostly corresponds to the transport of air mass from Northern Europe. To select days with intensive convection conditions, we used the mean diurnal solar fluxes at the surface as the parameter. Days with a high ratio of mean (24h) observed solar flux to mean clear-sky simulated by a simple radiative transfer model (Justus and Paris, 1985) at both sites as chosen. The threshold of 0.9 is used to select almost clear-sky conditions.

Potential confounding factors such as local topography, seasonal biomass burning episodes, and local wind recirculation were not explicitly controlled for in the analysis. The focus of this study was on capturing the overall variability of aerosol optical and microphysical properties between the urban and rural sites, rather than disentangling all possible contributing processes. Therefore, no additional filters were applied to remove specific meteorological or local biomass burning episodes.

## 3 Statistics of aerosol single-scattering properties at urban and rural site

**Fig. 2:**
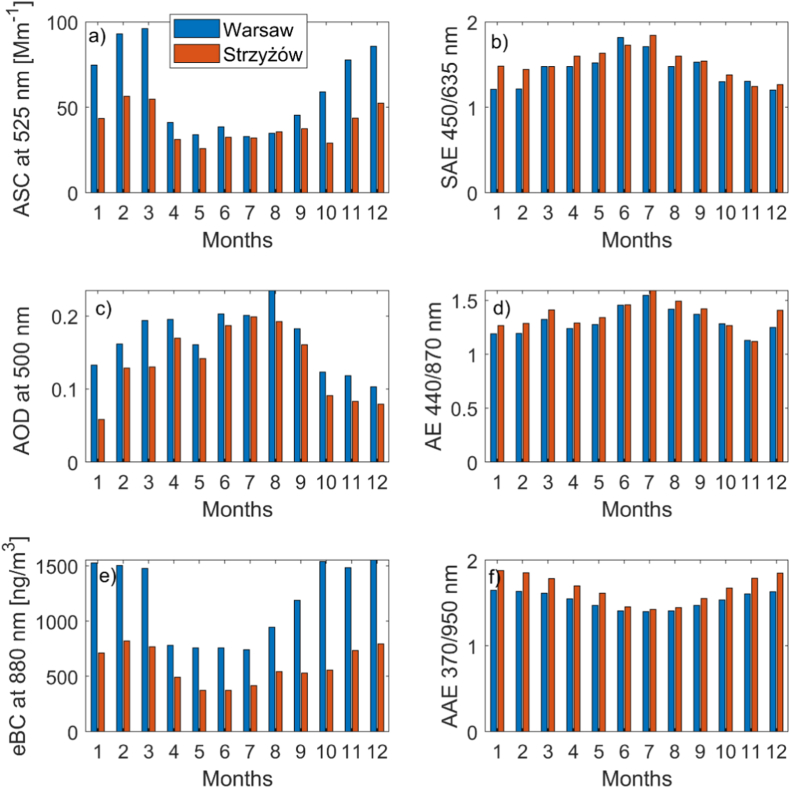
Monthly means of (a) ASC at 525 nm [Mm^−1^], (b) SAE (450/635 nm), (c) AOD at 500 nm, (d) AE (440/870 nm), (e) eBC at 880 nm [ng/m^3^], and (f) AAE in Warsaw (blue) and Strzyżów (red).

Fig. 2 illustrates that the monthly mean values of ASC at 525 nm are significantly larger (nearly twice) in winter compared to summer. Values from November to March are higher than those from April to October at both locations. The annual pattern of ASC reflects typical variability associated with increased urban and domestic pollutant emissions mostly from heating systems in autumn and winter (Szczepanik and Markowicz, 2018; Zgłobicki and Baran-Zgłobicka, 2024; Nidzgorska-Lencewicz and Czarnecka, 2015). The ASC is 33 % higher in Warsaw than in Strzyżów, with the difference reaching about 40 % in autumn and winter (see Table S1 in the Supplement). In contrast, during summer, the ASC in Warsaw and Strzyżów is significantly smaller (10 % difference). The means eBC and AAC are almost two time higher in city as at the rural site. Consequently, the SSA at 525 nm in Warsaw is significantly lower (0.84) compared to Strzyżów (0.89). The values measured at the Strzyżów site reflect background conditions, whereas the Warsaw site, located in the city center, is influenced by transport and other urban emissions. Conversely, the SAE values are higher in Strzyżów (especially in winter), suggesting that the effective radius of aerosol particles in Strzyżów is shifted toward the finer mode compared to Warsaw. Similarly, the AAE is higher in Strzyżów than in Warsaw, which suggests a stronger contribution of brown carbon (BrC) at the rural site, or a larger contribution of elemental carbon (EC) particles at the urban site. In addition, the annual cycle of AAE is opposite to that of SAE. The highest AAE values are observed during winter, when SAE reaches its minimum. In summer, the reduction in AAE accompanied by an increase in SAE can be explained by a decrease in the BrC-to-EC ratio.

In Warsaw, ASC and eBC are lower during the weekend (see the values in parentheses in Table S1 in the Supplement), by 5 % and 13 %, respectively. At the background site, the weekend effect is very small (1 % for ASC and 4 % for eBC). During winter, the reduction in aerosol extensive parameters is significantly larger at both sites. In Warsaw, ASC is 13 % lower and eBC is 21 % lower on Saturdays and Sundays. The SAE is also lower on weekends, consistent with the reduction in fine particle emissions. At the Strzyżów site, a measurable reduction in ASC (4 %) and eBC (10 %) is observed during weekends in the cold season. Columnar optical properties as AOD and AE (see Table S2 in the Supplement) show a significant difference between the sites. AOD at 500 nm is higher in Warsaw between 0.01 and 0.03. Such a difference is related not only to the megacity effect but also to the reduction of AOD with altitude. The Strzyżów site stands ∼200 m above the surrounding terrain. Assuming daytime well-mixed conditions in the lowest few hundred meters and mean near-surface extinction (ASC ≈ 50 Mm^−1^, AAC ≈ 7 Mm^−1^), the additional AOD associated with this local height difference is ∼0.01. This is a first-order estimate; actual values depend on PBL structure and vertical gradients of aerosol optical properties. Mean AE difference between Warsaw and Strzyżów is also negative, similar to surface SAE observations from the Aurora 4000 device.

**Fig. 3:**
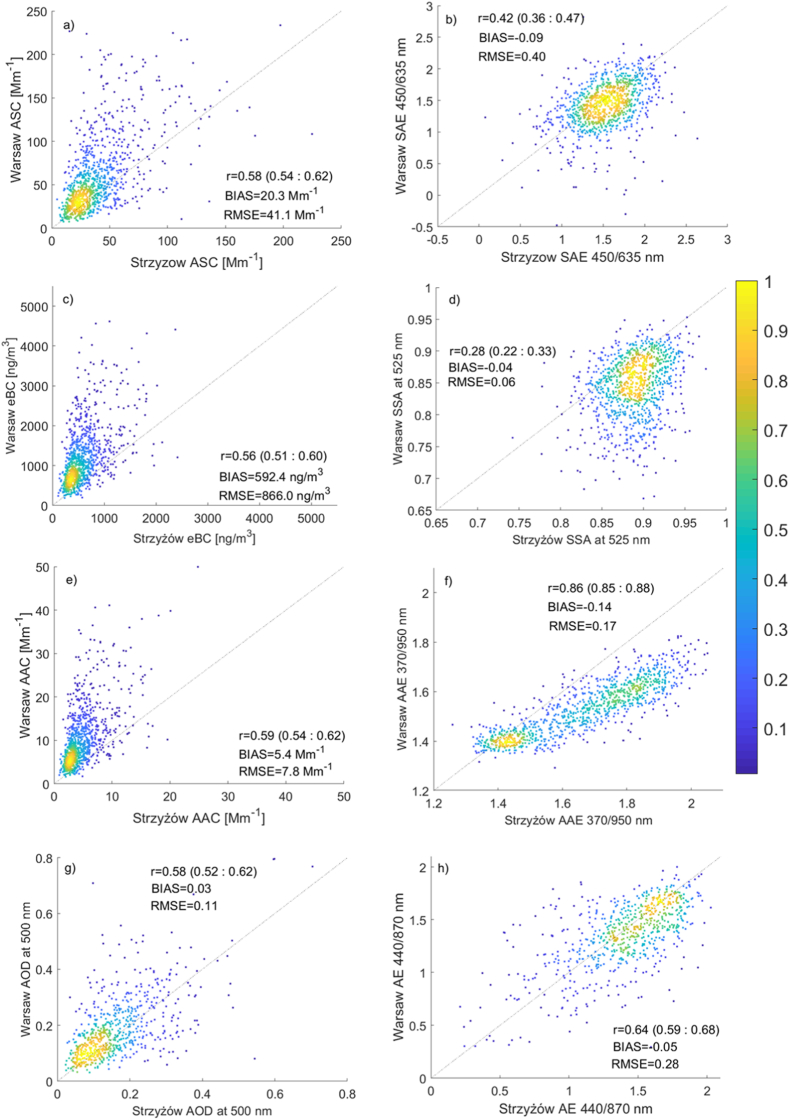
Pearson correlation coefficient for daily mean of (a) ASC at 525 nm [Mm^−1^], (b) SAE at 450/635 nm, (c) eBC at 880 nm [ng/m^3^], (d) SSA at 525 nm, (e) AAC at 525 nm [Mm^−1^] (f) AAE, (g) AOD at 500 nm, and (g) AE observed in Warsaw and Strzyżów. Color indicates the density of observations (normalized to the range 0–1), while black dotted lines represent the line of perfect agreement.

ASC and eBC are highly correlated. For example, the Pearson correlation coefficient in Warsaw is 0.83 (with a 95 % confidence interval of 0.81–0.85), and in Strzyżów it is 0.85 (with a 95 % confidence interval of 0.83–0.87). Summer ASC values are comparable at both locations, while winter values are higher in Warsaw. This difference can be attributed to changes in meteorological conditions and surface emissions between the warm and cold halves of the year. The annual cycle of eBC mirrors that of ASC, with a significant peak during the cold season and minimal values in summer. For the SAE and AE, the monthly mean difference at both locations is small in summer and significantly higher in the cold season (Fig. 3b–d). Winter and spring values in Warsaw are significantly lower than those in Strzyżów, suggesting that particles in Warsaw are larger compared to those in Strzyżów. The opposite pattern is observed in autumn. This discrepancy is likely related to variations in emissions throughout the year. In winter, emissions from heating systems dominate, while in the warm season, natural aerosols (such as those from fires or desert dust) can contribute significantly.

Long-term (2018–2025) statistics of AOD and AE at both sites (Fig. 2c and d) show an opposite annual cycle compared to the aerosol extensive optical properties at the surface. The explanation for this phenomenon provided by Szczepanik and Markowicz (2018) is related to the variability of meteorological conditions and the long-range transport of natural aerosols, mostly in the middle troposphere during spring and summer. In contrast, the significant reduction of PBL height and vertical mixing in winter leads to an increase in surface aerosol optical properties, while column-integrated properties remain unaffected. The mean AOD difference between Warsaw and Strzyżów is 0.02 at 500 nm. A similar value was found by Zawadzka et al. (2013) in their study comparing Warsaw with the Belsk rural site (45 km from the center of Warsaw), highlighting the urban effect on AOD.

Fig. 3 shows scatter plots for single-scattering optical properties measured at both sites. The mean daily aerosol properties in Warsaw and Strzyżów are moderately and highly correlated (correlation coefficient from 0.42 to 0.86) except the SSA, which has a very low correlation coefficient (0.29). What is interesting is that the Pearson correlation coefficient is only slightly higher for the columnar properties (AOD, AE) than for surface data (ASC, SAE). The AOD and AE should be related to the air mass properties, while the surface data can be contaminated by the local conditions (surface emission). In the background site, the surface aerosol properties are probably representative for a larger distance than in the city and.

therefore, background site data mostly describe the air mass. However, our data shows that even for Warsaw surface data, the correlation with Strzyżów is relatively high. In the case of the eBC, the correlation coefficient is slightly lower (0.56), but urban data has a high offset with respect to the background site.

**Fig. 4:**
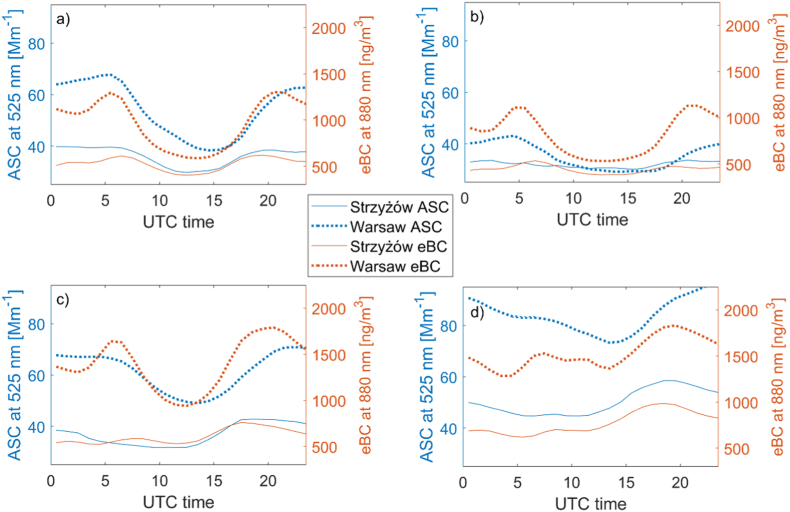
Mean diurnal variability of ASC (red) at 525 nm [Mm^−1^] and eBC (blue) at 880 nm [ng/m^3^] for Warsaw (dotted lines) and Strzyżów (solid lines) in (a) spring, (b) summer, (c) autumn, and (d) winter.

Fig. 4 shows the hourly average ASC and eBC during the four seasons. The diurnal cycle varies depending on the season and location. However, the diurnal pattern generally exhibits a minimum at both locations in the afternoon and a maximum at night. This pattern is influenced by the diurnal cycle of emission intensity and variations in meteorological conditions. Both the minimum and maximum are shifted to later hours in Warsaw compared to Strzyżów. In Strzyżów, the minimum occurs around 12 UTC and the maximum around 19 UTC, whereas in Warsaw, the minimum is at 14 UTC and the maximum at 23 UTC. This shift is likely due to the different sources of emissions, with heating systems being a significant factor in Warsaw and, to a lesser extent, transportation. In Warsaw, district heating systems provide heat and domestic water for residential consumers, offices, and businesses, primarily sourced from combined heat and power plants. In contrast, in Strzyżów and nearby towns, including areas close to Warsaw, heat is often obtained from individual households. This leads to higher emissions in the afternoon and evening in these areas. Consequently, the ASC maximum in Strzyżów occurs several hours earlier than in Warsaw. The mean difference between the maximum and minimum ASC over the diurnal cycle is greater in Warsaw (20 Mm^−1^) than in Strzyżów (8 Mm^−1^).

## 4 Impact of direction of air mass advection on aerosol optical properties

**Fig. 5:**
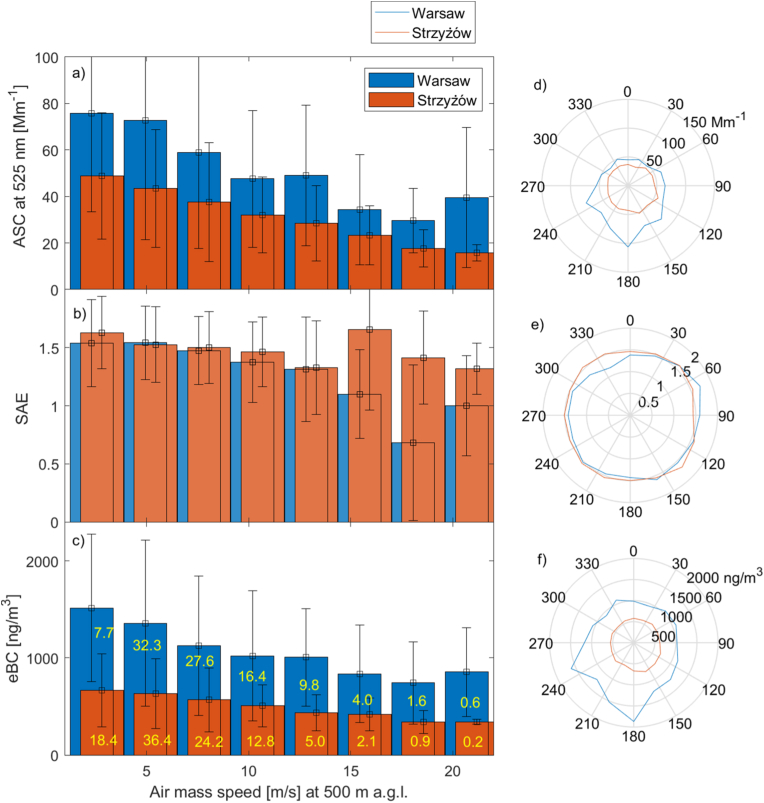
Variability of (a) ASC at 525 nm [Mm^−1^], (b) eBC at 880 nm [ng/m^3^], and (c) SAE in Warsaw (blue) and Strzyżów (red) as a function of speed [m/s] of air masses transported at 500 m a.g.l., based on HYSPLIT simulations. Error bars indicate the standard deviation, and yellow numbers indicate the relative contribution of cases in each bar [%]. Panels (d–f) correspond to the variability of ASC, SAE, and eBC with air masses azimuth.

This section provides information on the variability of aerosol single-scattering properties with respect to the direction of air mass flow. Aerosol single-scattering properties at both sites show sensitivity to the direction of air mass transport (Fig. 5d–f). In Warsaw, higher ASC is observed during circulation from the southern sector, while in Strzyżów, it is observed during southeast advection. During northern circulation (from west-northwest to northeast), the difference in ASC between Warsaw and Strzyżów is small (below 20 %). The variability of eBC with the direction of air mass transport is similar to that of ASC. Higher values are observed in Warsaw during southwestern transport, while in Strzyżów, they are mostly observed during southeastern circulation. In the case of the SAE, variability with the azimuth of air mass transport is small. Only for the northwest circulation in Warsaw and the east transport in Strzyżów is there a significant decrease in SAE measured. In Warsaw, this can be explained by the transport of large sea salt particles from the Baltic Sea and the North Sea. Columnar aerosol optical properties, such as AOD (see Fig. S3 in the Supplement), also show a peak in the south-eastern direction and non-significant changes due to air mass direction in the case of AE. The southeastern direction is occasionally associated with the transport of mineral dust or biomass particles in the middle atmosphere. On the other hand, the cleanest air mass is transported from the north-eastern direction.

Significant variability of aerosol properties has been found with air mass speed (Fig. 5a–c). Generally, extensive aerosol properties decrease significantly with air mass speed due to the reduction of air mass transformation over Poland. ASC is about 2–3 times higher during very slow transport in Warsaw and Strzyżów compared to cases when the transport is fast. Similarly for eBC. However, the number of cases with very fast transport (more than 15 m/s, last 3 bars in Fig. 5a) is very small, and therefore the mean values are subject to large uncertainties. In the case of SAE, faster transport is associated with a reduction of fine-mode particles. Air mass transformation over Poland is responsible for the increase in SAE due to the emission of fine-mode particles. Stronger wind cases may also be related to the passage of atmospheric fronts and precipitation events, which enhance wet deposition and contribute to the observed reduction of aerosol load.

Table 1 shows the Pearson correlation coefficient, bias, RMSE, and mean value of ASC at 525 nm at the Warsaw and Strzyżów sites for west, east, north, and south advection. We found that during northern circulation (mostly Arctic air masses), the mean bias between Warsaw and Strzyżów ASC is small (less than 6.0 Mm^−1^ during the day and 10.2 Mm^−1^ during the night). In contrast, during transport from Southern Europe, the mean bias is very large (42.4 Mm^−1^ during the day and 59.7 Mm^−1^ during the night). In the case of western and eastern transport, the mean bias is similar (15.8–17.2 Mm^−1^ during the day and 25.6–29.8 Mm^−1^ during the night). Therefore, such values can be assumed as typical differences between urban and background sites (urban effect). Pearson correlation coefficient between ASC in Warsaw and Strzyżów changes from 0.37 for the southern direction to 0.69 for the northern direction. In the case of the eBC (Table 1) the correlation varies from 0.27 for the southern direction to 0.72 for the northern direction. There is a significant difference between the day's and night's values. During the night, the correlation coefficient between both sites is lower for all air mass directions except the northern sector. Similar to ASC, also for eBC, the higher bias between Warsaw and Strzyżów is observed at southern circulation (787 ng/m^3^ during the day and 1346 ng/m^3^ during the night). However, for the northern flow, the bias is not negligible as for the ASC. During the day, the mean eBC bias is 279 ng/m^3,^ while during the night it is 524 ng/m^3^. Thus, the impact of Warsaw emissions on eBC is significantly higher than for ASC. The analysis revealed that all correlations reached statistical significance at the 95 % confidence level.

**Table 1:** Pearson correlations coefficient, rmse, bias (Warsaw-Strzyżów), and mean values of the aerosol scattering coefficient at 525 nm [Mm^−1^] (upper values) and the equivalent black carbon concentration at 880 nm [ng/m^3^] (lower values) for Warsaw and Strzyżów site for inflow from four geographic directions during night (18-06 UTC) and day (06–18 UTC).

Advection	Hour	r	rmse	bias	Warsaw	Strzyżów
All data	day	0.57	39.9	17.0	54.1	37.1
		0.58	782	461	1035	574.3
	night	0.47	54.8	28.3852	67.7	39.3
		0.37	1408		1467	615
West	day	0.55	39.2	15.8	49.6	33.8
		0.52	800	488	1023	535
	night	0.46	54.0	25.1	63.7	36.5
		0.35	1337	857	1426	569
East	day	0.53	37.8	17.2	59.0	32.8
		0.68	506	355	929	574
	night	0.57	50.0	29.8	73.5	43.8
		0.36	1189	757	1382	625
North	day	0.70	28.1	6.0	36.5	30.5
		0.72	590	279	777	498
	night	0.65	37.2	10.2	46.7	36.5
		0.62	1157	524	1122	598
South	day	0.56	58.7	42.4	83.8	41.3
		0.56	1013	787	1429	642
	night	0.45	79.0	59.7	100.4	40.8
		0.27	1915	1346	2007	661

For the analysis of air mass transformation over Poland, we select cases when the air mass passes both sites (see section 2.4). Transport from the southern direction is slightly faster than from the opposite direction. The average air mass transport speed at 500 m from Strzyżów to Warsaw is 7.3 m/s during the day and 7.4 m/s during the night, while from Warsaw to Strzyżów it is 6.0 m/s during the day and 6.2 m/s during the night. Table 2 summarizes the mean aerosol optical properties during both types of air mass advection. For the type N (Arctic air mass), we observed a higher correlation coefficient for ASC in Warsaw and Strzyżów than for type S (tropical air mass). Also, the mean difference is very small (2.7 Mm^−1^ during the day) for type N. Only during the night, the bias is slightly higher (6.3 Mm^−1^). During transport from the southern direction (from Strzyżów to Warsaw), these mean differences increase due to pollution emissions and air mass transformation (mean bias is 27.4 Mm^−1^ in day and 42.3 Mm^−1^ in night). In the case of Arctic air masses, the difference between Warsaw and Strzyżów is almost negligible (during the day), which means that the transformation of the air mass between the two sites (a distance of 265 km) close to the mean urban effect. Similar changes are observed for columnar data (AOD at 500 nm). For the N-type, the mean bias is only 0.007 (5 %) for S-type is 0.052 (34 %). Different results are obtained for the eBC. For both types of flow, the mean bias is very large (56 % for S and 45 % for N during the day), probably due to the very high emission of absorbing particles from the transport sector in Warsaw city. Note that during nighttime and S-type circulation, the correlation coefficient of eBC between Warsaw and Strzyżów is not statistically significant. A similar lack of significance is observed for SAE during nighttime N-type circulation. For the S flow the ASC in Warsaw $\sigma_{wars}^{S}$ can be written as:

(3) $$\sigma_{wars}^{S}=\sigma_{strz}^{S}+\Delta_{t}+\Delta_{c}$$

where $\sigma_{strz}^{S}$ corresponds to ASC in Strzyżów during S-flow, ${\;\Delta }_{t}$ and $\Delta_{c}$ are the change of ASC due to air mass transformation between Warsaw and Strzyżów and change of ASC in the city, respectively. Similarly for the N flow the ASC in Strzyżów $\sigma_{strzy}^{N}$ can be expressed by

(4) $$\sigma_{strzy}^{N}=\sigma_{wars}^{N}+\Delta_{t}-\Delta_{c}\text{.}$$

**Table 2:** Pearson correlations coefficient, number of cases, rmse, bias (Warsaw-Strzyżów), mean values and standard deviation of the ASC at 525 nm [Mm^−1^], SAE (450/635 nm), eBC at 520 nm [ng/m^3^], AOD at 500 nm, and AE at 440/870 nm for Warsaw and Strzyżów when back trajectories passes both site during night (18-06 UTC) and day (06–18 UTC) in the radius less than 100 km.

Variable	Direction	Hour	r	#n	rmse	bias	Warsaw	Strzyżów
ASC	S	day	0.52	93	49.3	27.4	70.1 ± 43.8	42.7 ± 38.4
		night	0.32	89	64.8	42.3	83.8 ± 49.2	41.5 ± 30.4
	N	day	0.76	96	24.1	2.7	38.9 ± 36.9	36.3 ± 28.2
		night	0.59	93	37.8	6.3	50.1 ± 45.6	43.9 ± 31.1
SAE	S	day	0.63	93	0.26	0.00	1.51 ± 0.30	1.51 ± 0.30
		night	0.44	89	0.35	0.012	1.52 ± 0.30	1.53 ± 0.35
	N	day	0.28	96	0.59	−0.16	1.31 ± 0.52	1.47 ± 0.42
		night	0.21	93	0.81	0.01	1.40 ± 0.64	1.39 ± 0.65
eBC	S	day	0.57	97	806	580	1299 ± 680	640 ± 427
		night	0.20	96	1650	1108	1720 ± 1237	612 ± 361
	N	day	0.71	99	472	194	768 ± 574	574 ± 374
		night	0.48	98	851	384	1061 ± 865	444.4 ± 429
AAE	S	day	0.86	97	0.29	−0.27	1.32 ± 0.12	1.59 ± 0.17
		night	0.64	96	0.30	−0.26	1.39 ± 0.14	1.65 ± 0.18
	N	day	0.79	99	0.29	−0.27	1.27 ± 0.10	1.54 ± 0.16
		night	0.71	98	0.26	−0.23	1.34 ± 0.13	1.57 ± 0.17
AOD	S	day	0.71	63	0.094	0.052	0.205 ± 0.110	0.153 ± 0.086
	N	day	0.54	89	0.068	−0.007	0.128 ± 0.068	0.135 ± 0.073
AE	S	day	0.78	63	0.26	0.00	1.27 ± 0.37	1.27 ± 0.40
	N	day	0.60	89	0.28	−0.13	1.34 ± 0.28	1.46 ± 0.29

Solving of this system of equations we can estimate the air mass transformation and urban effect as follow:

(5) $$\Delta_{t}=\frac{1}{2}\left[\left(\sigma_{wars}^{S}-\sigma_{strz}^{S}\;\right)-\left(\sigma_{wars}^{N}-\sigma_{strz}^{N}\;\right)\right]=\frac{1}{2}\left[{BIAS}^{S}-{BIAS}^{N}\right]$$

(6) $$\Delta_{c}=\frac{1}{2}\left[\left(\sigma_{wars}^{S}-\sigma_{strz}^{S}\;\right)+\left(\sigma_{wars}^{N}-\sigma_{strz}^{N}\;\right)\right]=\frac{1}{2}\left[{BIAS}^{S}+{BIAS}^{N}\right]$$

where the BIAS^N^ and BIAS^S^ corresponds to mean Warsaw - Strzyżów bias during N and S flow type. For the ASC the ${\;\Delta }_{t}$ is 12.4 ± 5.2 Mm^−1^ and 18.0 ± 5.5 Mm^−1^ during day and night, respectively. While the urban effect is 15.1 ± 5.2 Mm^−1^ during day and 24.3 ± 5.5 Mm^−1^ during night. Similar calculation for eBC provides following results. The air mass transformation (${\;\Delta }_{t}$) is 193 ± 59 ng/m^3^ during day and 1362 ± 86 ng/m^3^ during night and 387 ± 59 and 746 ± 86 ng/m^3^ for megacity effect. For the AOD emission between Warsaw and Strzyżów increase this quantity by 0.029 ± 0.009 and $\Delta_{c}$ is 0.023 ± 0.009. This value is slightly higher (0.02) than estimated by Zawadzka et al. (2013) based on sun photometer measurements in Warsaw and Belsk rural stite (45 km from Warsaw).

Additionally, the SAE and AE values slightly increase during air mass transformation over Poland, which corresponds to fine particle emissions from various sectors. For the S flow, the columnar AE is identical (1.27) at both sites. Similarly, the surface SAE shows the same values at both sites, increasing from 1.51 during the day and 1.52–1.53 at night. Significantly higher surface SAE than columnar AE for the S flow can be partly explained by the mineral dust particles, which are occasionally observed during such air mass advection. Such episodes are usually observed in the middle troposphere (Szczepanik et al., 2023), so they impact on columnar aerosol properties but not for surface aerosol optical properties. In the case of the N flow, the SAE change is negligible at night and significantly increases from Warsaw to Strzyżów during the day (1.31–1.47). For the columnar data (AE) increases from 1.34 in Warsaw to 1.46 in Strzyżów.

## 5 Impact of convection on aerosol single-scattering properties

**Fig. 6:**
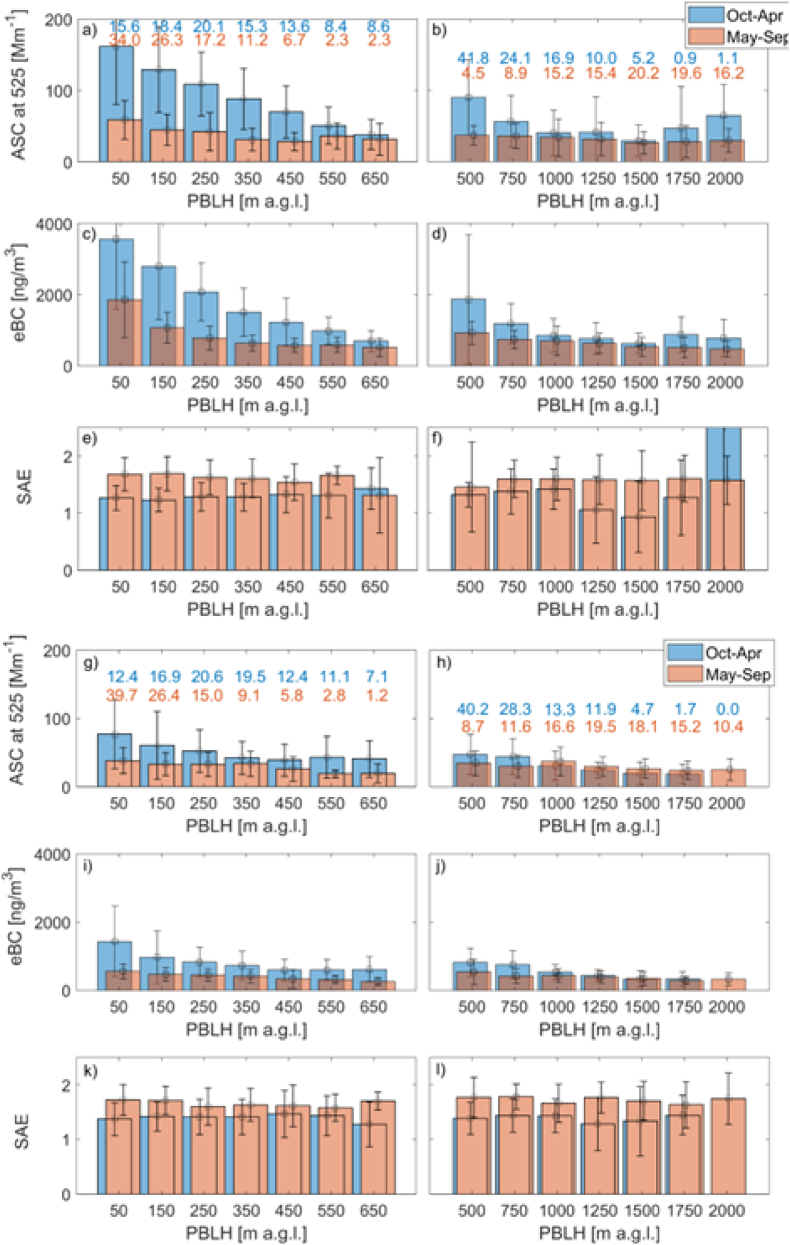
Surface ASC at 525 nm [Mm^−1^] (a, b), eBC at 880 nm [ng/m^3^] (c, d), and SAE (e, f) a function of the PBLH [m a.g.l.] obtained from the ERA5. Panels (a, c, e) correspond to Warsaw site at 00 UTC, and panels (b,d, f) to Warsaw at 12 UTC. Panels (g, i, k), and (h, j, l) show the respective results for Strzyżów site at 00 and 12 UTC. Blue bars indicate the October and April period, while orange bars indicate the May–September period. The first and last bins (lowest and highest PBLH) include all cases with PBLH values smaller or larger than the defined thresholds. The blue and orange numbers in panels a,b,g, and f correspond to frequency PBLH bins, respectively, for October–April and May–September months.

The surface extensive aerosol optical properties depend on vertical mixing and PBLH. Systematic plot (Fig. 6) shows surface ASC, eBC and SAE variability with PBLH (based on the ERA5) at 00 and 12 UTC for Warsaw (Fig. 6a–f) and Strzyżów (Fig. 6g–l) site, respectively. The presented data are divided into two periods cold (October–April, blue bars) and warm (May–September, orange bars) to separate emissions from the heating systems during the cold season. During the night (00 UTC), the ASC is significantly enhanced with reduced PBLH. For example, in Warsaw (cold season) ASC at 00 UTC is reduced 4.5 times when PBLH is increased from 50 to 650 m. Up to 3 times reduction is observed at 12 UTC when PBLH is increased from 500 to 15000 m. In contrast, the reduction of ASC with increasing PBLH is smaller during the warm season; however, still significant. In the case of eBC, the relationship is similar to that for ASC. For example, the reduction of eBC is close to 5.5 times during the cold season at 00 UTC when PBLH increases from 50 to 650 m and about 3 times at 12 UTC when PBLH increases from 500 to 1500 m. During the warm season sensitivity of eBC due to PBLH variability is also smaller than in the cold period. There is no systematic variation in the case of the SAE and PBLH. Some observed fluctuations are probable due to the statistical representation of data. For the background site, the ASC and eBC show similar variation with PBLH to the Warsaw site. However, the largest reduction of both parameters is noted in the cold season at 00 UTC (Fig. 6a–c). Also, for the SAE, no significant variation with PBLH is observed. Similar analysis for the whole period shows a significant reduction of SEA with PBLH, but it is due to the annual cycle of SAE and PBLH.

**Fig. 7:**
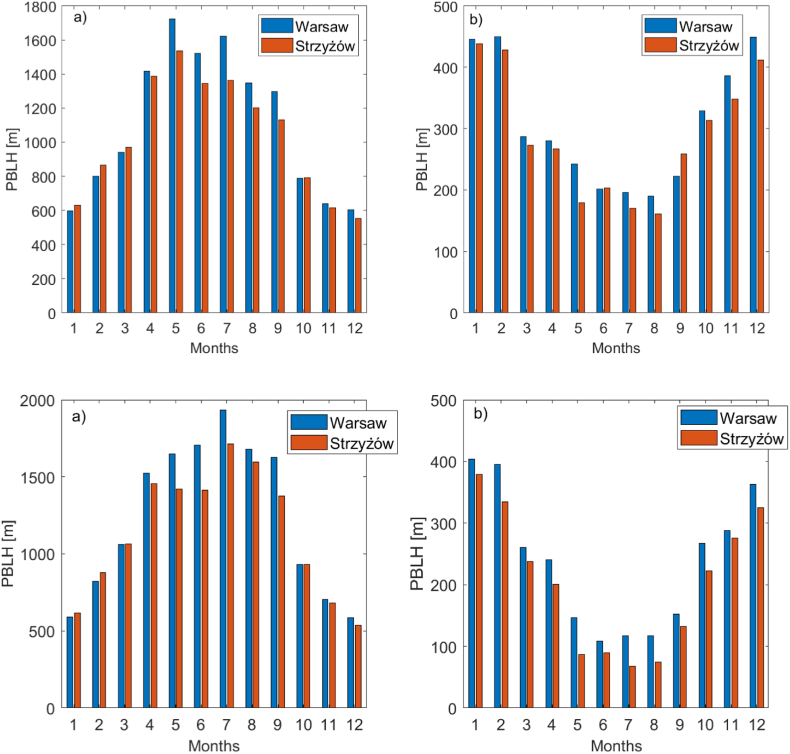
Monthly mean PBL height [m] in Warsaw (blue) and Strzyżów (red) at (a) 12 UTC and (b) 00 UTC, obtained from the ERA5.

The noon PBLH exhibits a typical annual cycle (Fig. 7), reaching a maximum of around 1500–1700 m (in Warsaw) during spring and summer, and a minimum during late autumn and winter (around 600 m). However, the PBL in spring and summer is significantly deeper in Warsaw than in Strzyżów (by approximately 50–200 m), which is really likely due to the urban heat island effect, but in the case of ERA5, and meteo data with spatial resolution of 0.25x0.25^o^ such local-scale effect may not be fully resolved. At midnight, the annual cycle is reversed, with the maximum PBLH occurring in winter and the minimum in summer. The nighttime PBLH is primarily influenced by surface radiative cooling and geostrophic wind. During winter nights, the PBL becomes higher due to increased turbulence and reduced radiative cooling. The first effect is caused by a higher pressure gradient, while the second is due to a greater cloud fraction compared to summer. The next conclusion from Fig. 7 is the diurnal variability of PBLH, which in the warm season is very large (typically from 200 m at night to 1900 m close to noon) and very small in the cold season (amplitude between day and night about 150–200 m). Similar results are reported by Julaha et al. (2025) based on numerical models, re-analysis, and ceilometer observation in Košetice (Czech). For the Warsaw site, these model-derived values are similar to the long-term average (2008–2018) of lidar/ceilometer-derived PBLH (Wang et al., 2020). The pronounced diurnal variability of the PBLH in spring and summer, compared to the more stable (less vertical mixing) winter conditions, is responsible for the high diurnal variability in aerosol extensive parameters during the warm season.

Table S4 in the Supplement shows the variability of the PBLH based on the direction of air mass inflow. The highest daily PBLH was observed for air masses arriving from Northern Europe, while the lowest was from the south direction. Interestingly, during northern circulation (mostly Arctic cold air masses), the noon PBLH is significantly higher (1303 m in Warsaw and 1197 m in Strzyżów) than during southern circulation of warm tropical air masses (821 m in Warsaw and 851 m in Strzyżów). In summer, the mean PBLH during northern circulation reaches 1589 m in Warsaw and 1390 m in Strzyżów, whereas for southern transport, it is 1195 m and 1164 m in Warsaw and Strzyżów, respectively. These results are consistent with the variability of surface sensible heat flux (based on the eddy covariance method) with the direction of air mass advection in Warsaw (see Fig. S5 in the Supplement), which is a key driver controlling the PBLH. In the case of tropical air masses, the heat flux is nearly half that of Arctic air masses. Thus, pollution during the day can be more efficiently diluted during northern transport compared to southern advection. Additionally, air masses from northern Europe are relatively cleaner (both in terms of AOD and surface optical properties) than air masses from southern Europe.

**Fig. 8:**
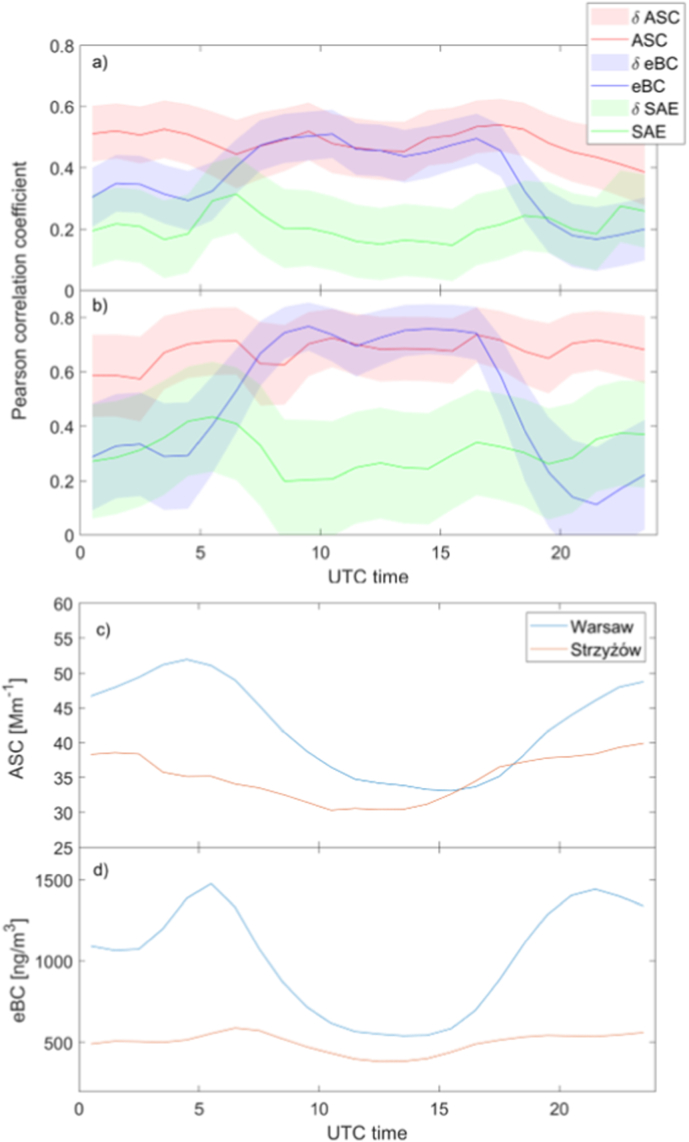
Diurnal variability of the Pearson correlation coefficient for ASC, eBC, and SAE measured at both sites during warm season (May–September): (a) all data and (b) high-insolation (mostly clear-sky) days. Shading represents the 95 % confidence interval of the correlation coefficient. The panel (c) and (d) show the mean diurnal variability of ASC at 525 nm [Mm^−1^] and eBC at 880 nm [ng/m^3^] in Warsaw (blue) and Strzyżów (orange) for high-insolation (mostly clear-sky) days.

Fig. 8a–b shows the diurnal variability of the Pearson correlation coefficient for ASC, eBC, and SAE measured in Warsaw and Strzyżów during the warm season (May–Sep). For all days (Fig. 8a), the correlation coefficient slightly increases (up to 0.55) during the daytime for the extensive parameters (ASC, eBC). When days with high solar radiation flux are selected (mean surface flux higher than 90 % of the mean clear-sky flux), the correlation coefficient increases up to 0.75 for ASC and up to 0.8 for eBC. In the case of eBC, the correlation coefficient is very low at night (less than 0.3), while for ASC, the value is moderate (approximately 0.4–0.5). For SAE, the correlation coefficient at both sites is negligible during both day and night. The very low daytime correlation coefficient can be explained by high uncertainty. SAE uncertainty increases with a decrease of ASC (Markowicz and Chiliński, 2020). Convection mixing is responsible for the high correlation coefficient for ASC and eBC at both locations. The difference in ASC between 10 and 20 UTC is very small (less than 5 Mm^−1^, see Fig. 8c–d). We found that from May to September during 59 % days ASC difference between Warsaw and Strzyżów is less than 5 Mm^−1^ at 12 UTC. In the case of all days 43 % data shows a difference of less than 5 Mm^−1^. For the eBC the minimal difference of 200–250 ng/m^3^ is observed between 10 and 16 UTC. What is interesting is that the afternoon peak in transportation in Warsaw is not detected by both ASC and eBC data. It is probably due to intensive convective mixing close to 15–17 LT (13–15 UTC). The morning peak in eBC and ASC at 07 UTC (09 LT) is observed. It can be explained by the morning traffic maximum and not yet well-developed PBL. Therefore, we can conclude that in the case of the ASC intensity of aerosol emission has a negligible effect on the surface ASC during intensive convection conditions. However, in the case of the eBC the urban effect (emissions) during such weather conditions is also observed. Unfortunately, we do not have independent emission estimates that would allow for partial correlation or regression analysis to separate these effects. Therefore, our interpretation is limited to the observation that diurnal co-variability is consistent with PBL mixing, while the specific contribution of emissions cannot be quantitatively isolated.

## 6 Summary and conclusion

In this study, three years (2022–2025) of surface and seven years (2018–2025) of columnar aerosol single-scattering optical data collected at an urban (Warsaw) and a background (Strzyżów) site are analyzed. Aerosol scattering coefficient (ASC) at 525 nm in Warsaw (59.2 ± 43.2 Mm^−1^), on average, is 34 % higher than in Strzyżów (39.3 ± 25.0 Mm^−1^) in the Subcarpathian region, while equivalent black carbon (eBC) mass concentration in Warsaw (1182 ± 753 ng/m^3^) is higher by 100 % than in Strzyżów (590 ± 335 ng/m^3^). Also, the aerosol optical depth (AOD) at 500 nm is higher in Warsaw (0.185) than in Strzyżów (0.159), however, the change of AOD between both sites is only 14 % (significantly smaller than for ASC or eBC). This is because AOD represents the integrated aerosol load of the whole atmospheric column (air mass properties), whereas ASC and eBC reflect conditions in the lower, near-surface troposphere. Ramachandran and Ansari (2025) showed that the contribution of eBC to AOD is generally ≤10 % globally, indicating that its role in shaping columnar optical depth is secondary. Spectral dependence of both ASC and AOD, quantified by surface scattering Ångstrom exponent (SAE) and columnar Ångstrom exponent (AE), shows higher values at the background site (SAE of 1.52, AE of 1.41) in comparison to the urban location (SAE of 1.43, AE of 1.36). The consequence of larger differences in eBC than ASC is the fact that the mean surface single-scattering albedo (SSA) in Warsaw is significantly lower (0.84) than in Strzyżów (0.89) site. Studies comparing urban and peri-urban (or rural) locations have consistently shown enhanced aerosol absorption and optical depth in urban centers. For instance, Paris exhibited a ∼0.1 decrease in SSA and an increase of ∼0.01 in the imaginary part of the refractive index in the city compared with peri-urban and forested sites (Di Antonio et al., 2025). Madhavi Latha and Badarinath (2004) also reported a reduction in SSA from 0.85 to 0.76 between rural (Srisailam) and urban (Hyderabad) environments in India. In North China Plain megalopolises, AOD decreased and SSA slightly increased toward suburban areas (AOD ∼0.43–0.86; SSA ∼0.89–0.93; Zheng et al., 2020).

Similar Pearson linear correlation coefficients between ASC (0.58) and AOD (0.58) observed at both sites may suggest that urban impact is relatively small and surface as well as columnar optical properties are mostly controlled by the air mass properties (Zdun et al., 2016) and vertical mixing rather than by local emission. In case of the dominant role of local (i.e., urban) emissions on aerosol properties, the correlation coefficient for AOD should be significantly higher than for surface ASC. Based on the difference in aerosol optical properties at both sites during two types of circulation (when the air mass crosses both sites), the effect of urban and air mass transformation was estimated. During transport of air mass between Warsaw and Strzyżów site (a distance of 265 km), the ASC, AOD, and eBC increase respectively by 13.3 Mm^−1^ (39 %), 0.029 (18 %), and 191 ng/m^3^ (32 %). The increase of aerosol properties in Warsaw due to local emission is estimated at 19.7 Mm^−1^ (32 %), 0.023 (15 %), and 512 ng/m^3^ (43 %). Previous research by Di Antonio et al. (2023), based on the Multi-Angle Implementation of Atmospheric Correction (MAIAC) product of AOD, reported enhancements of AOD due to the urban emission with respect to regional AOD by 57 %, 55 %, 39 % and 32 % for large cities of Barcelona, Lisbon, Paris, and Athens, respectively. However, negative impacts are observed for Amsterdam (−17 %) and Brussels (−6 %) due to higher background emissions. Similar satellite-based analyses were performed for the Moscow megacity using the same data product by Zhdanova et al. (2020). Although MAIAC data show good agreement with AERONET observations and capture urban–background gradients, the satellite retrievals tend to mask the urban effect. Ground-based AERONET measurements indicate that the annual median AOD difference between Moscow and the suburban area varies from −0.002 to +0.03, with a statistically significant positive bias in most years and an average difference of about 0.02 (Chubarova et al., 2011). According to Papachristopoulou et al. (2022), most cities (∼60 %) exhibit higher mean AOD over their urban areas, with Chinese and Indian megacities generally showing elevated AOD in surrounding regions. Shanghai shows the largest urban–surrounding difference (13 %), though in many cities this difference is smaller due to already high urban AOD.

Monthly mean of surface extensive parameters (ASC, eBC) in the cold season are much higher in Warsaw than in Strzyżów, indicating higher emissions related to heating systems (Zgłobicki and Baran-Zgłobicka, 2024). In the warm season, the values are comparable (for the ASC), which on the one hand may be due to lower emissions in the city, while on the other hand, to more intensive convective mixing during spring and summer days. The influence of convection mixing is indicated by diurnal variations of ASC and eBC parameters. The ASC and eBC at the two sites are clearly separated at night (with higher values in Warsaw) while they are comparable during the day. During the warm season, 59 % of observations of ASC close to noon time show the difference between Warsaw and Strzyżów site less than 5 Mm^−1^. Therefore, analysis of convective conditions confirmed the influence of vertical mixing in the atmospheric boundary layer on the level of air pollution near the ground. In spite of high traffic emissions in Warsaw, the air quality at this site during spring and summer days is similar to that observed at the mountain background site in Strzyżów, though this comparison is specific to these studied locations and conditions.

A significant variability of ASC and eBC with PBLH at both sites has been found. Higher reduction of ASC and eBC with the increase of PBLH is observed during noon (due to convection mixing) than at midnight. Previous research by Murthy et al. (2020) in Delhi shows that the PM_2.5_ mass concentration decreased by 14, 13, and 7 μg/m^3^ in December, January, and February, respectively, for every 100 m increase of PBLH. Schäfer et al. (2006) reported that the correlation coefficient of pollutant concentrations with PBLH is smallest inside street canyons. In addition, correlations are larger for the urban sites than for the rural sites and higher in winter than in summer. Salvador et al. (2020) show that a decrease in the PBLH in Madrid is associated with a linear increase in the number of days with the air pollution exceedances of EU norms. However, the effect of PBLH on surface aerosol concentration and particle optical properties is more complicated due to the hygroscopic effect. Therefore, it requires more sophisticated future study.

These findings provide observational constraints for air quality models, particularly emphasizing the role of boundary layer dynamics and regional transport in shaping aerosol properties. They also indicate that mitigation measures should primarily target heating-related emissions in the cold season, when their impact on air quality is strongest.

## CRediT authorship contribution statement

**S. Kłapiński:** Writing – review & editing, Writing – original draft, Visualization, Software, Formal analysis, Data curation. **K.M. Markowicz:** Writing – review & editing, Writing – original draft, Supervision, Methodology, Conceptualization. **I.S. Stachlewska:** Writing – review & editing, Resources, Methodology, Funding acquisition.

## Declaration of competing interest

The authors declare that they have no known competing financial interests or personal relationships that could have appeared to influence the work reported in this paper.

## Data Availability

Data will be made available on request.
