# Supplementary material for: Impact of atmospheric condition on aerosol optical properties in urban and rural environment
Source: Atmos Pollut Res. 2026 May;17(5):None. doi: 10.1016/j.apr.2025.102884 (PMC13097985; doi:10.1016/j.apr.2025.102884)
Supplement: Multimedia component 1 [file mmc1.docx]

**Supplementary Material**

**Impact of atmospheric condition on aerosol optical properties in urban and rural environment.**

**Kłapiński, S., K.M. Markowicz, I.S. Stachlewska**

University of Warsaw, Faculty of Physics, Institute of Geophysics, Pasteura 5, 02093 Warsaw, Poland


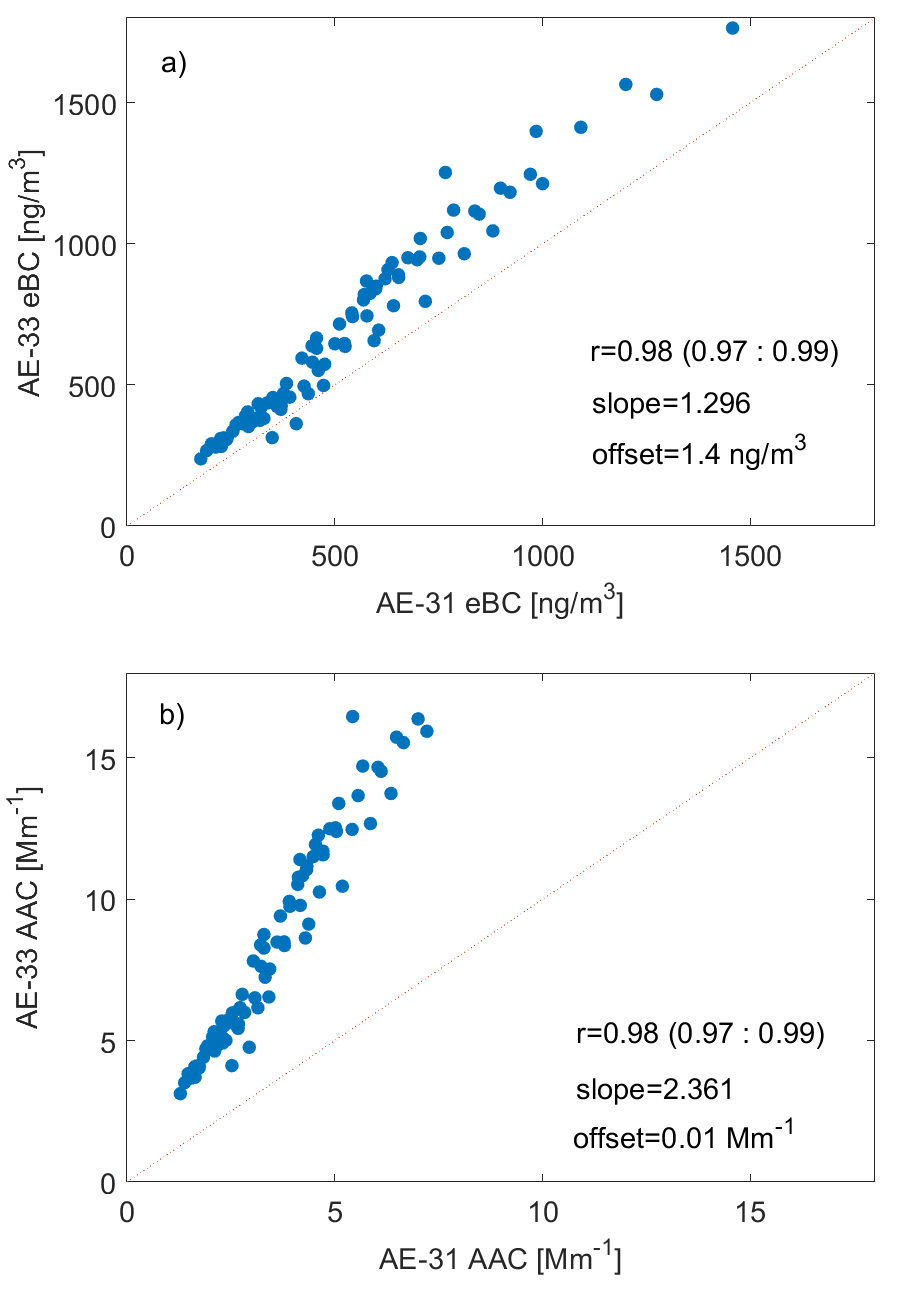


Fig. S1 Scatter plot of the (a) eBC and (b) AAC at 520 nm obtained from AE-33 and AE-31 during intercomparison experiment in Warsaw (July 2024). Dotted line shows perfect agreement.


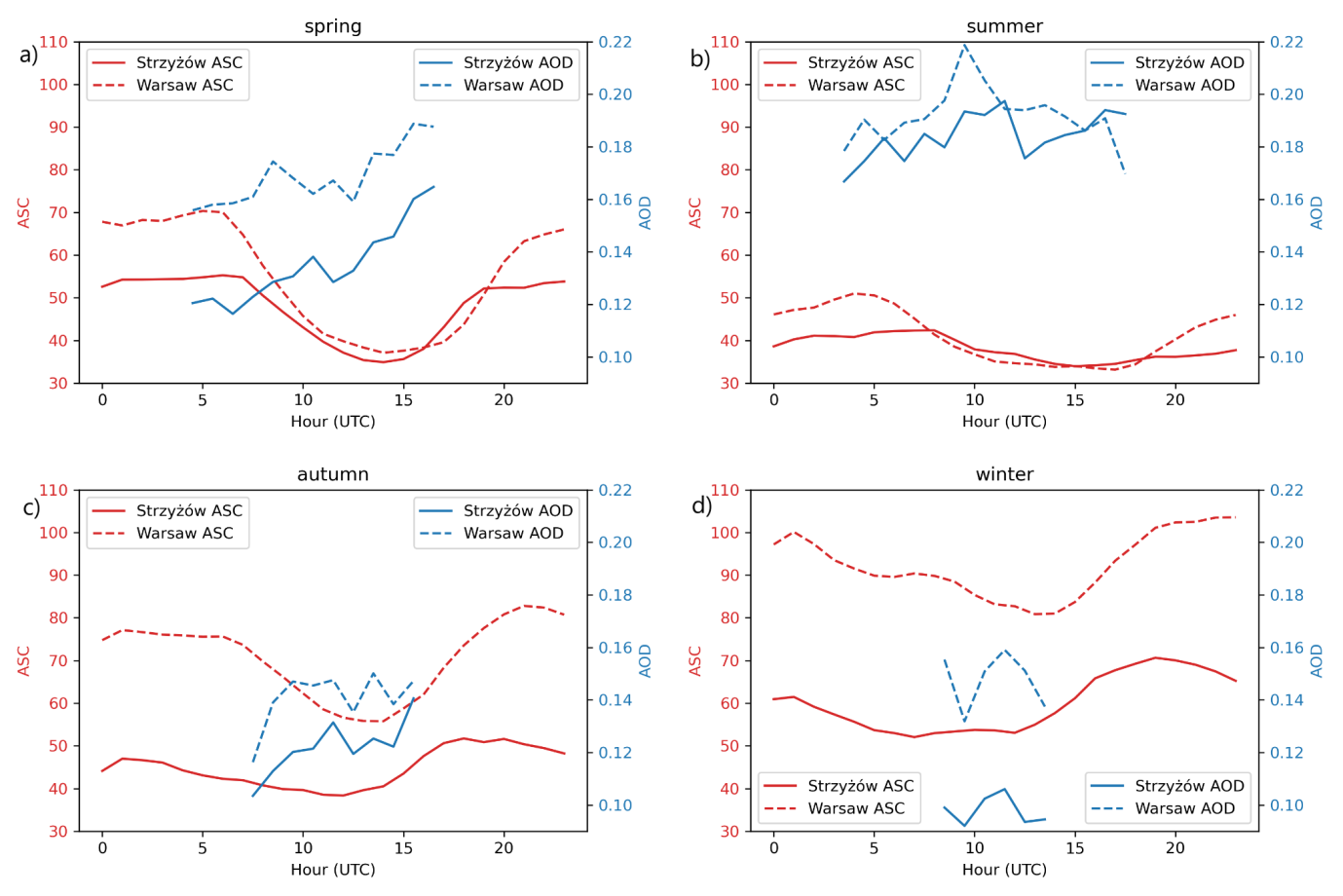


Fig. S2 Mean diurnal variability of ASC at 525 nm [Mm^-1^] and AOD at 500 nm for Warsaw (dotted line) and Strzyżów (solid line) in (a) spring, (b) summer, (c) autumn, and (d) winter.


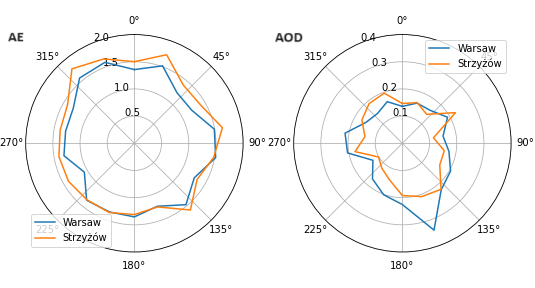


Fig. S3 Polar plot for AE and AOD for Warsaw (blue) and Strzyżów (orange) based on sun photometer and nephelometerobservation and HYSPLIT simulation at 500 m a.g.l.


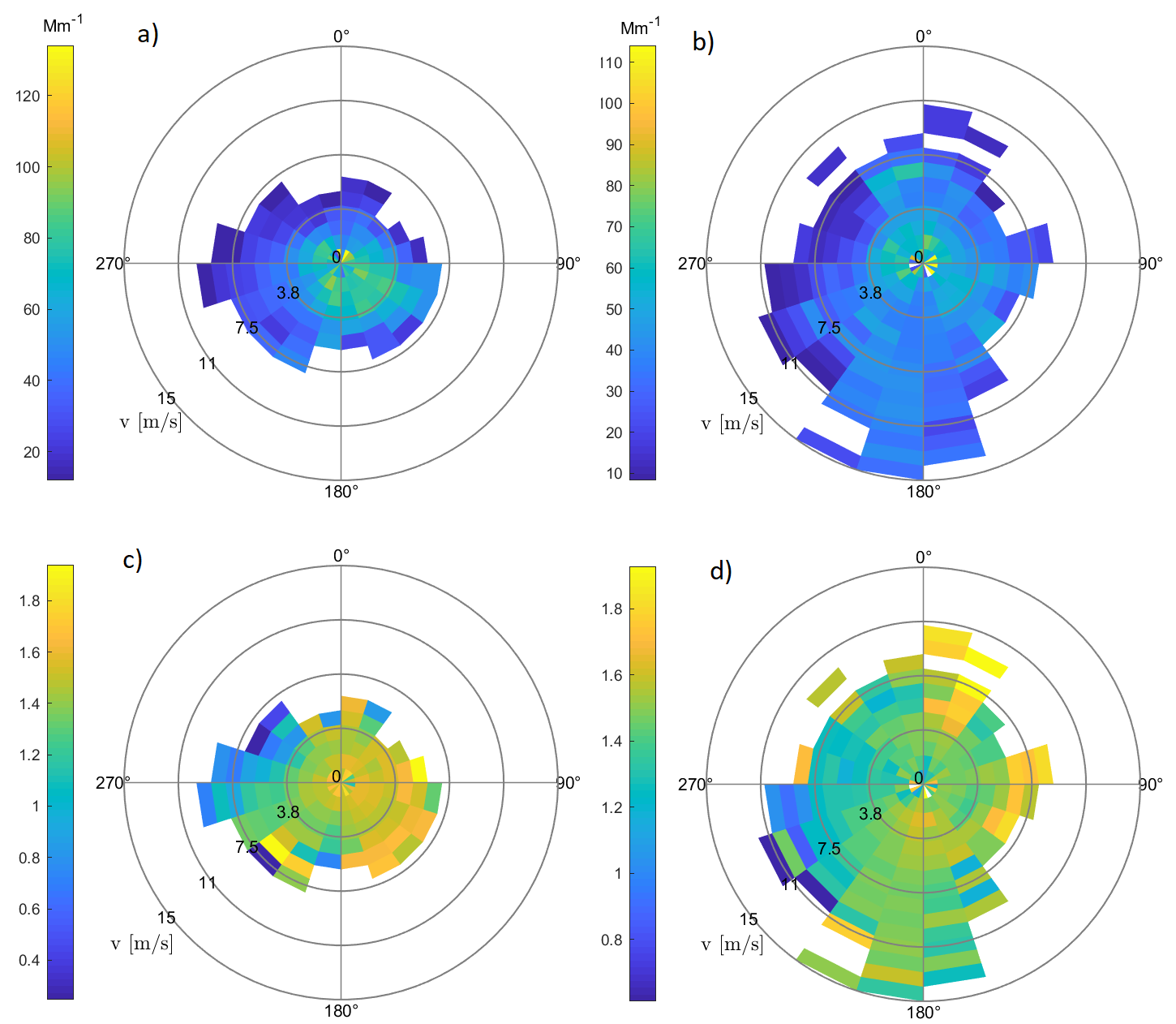


Ryc. S4 Dependence of ASC at 525 nm [Mm^-1^] (a and b) and SAE (c and d) on wind direction and wind speed [m/s] (colors) in Warsaw (a,c) and Strzyżów (b,d).

**
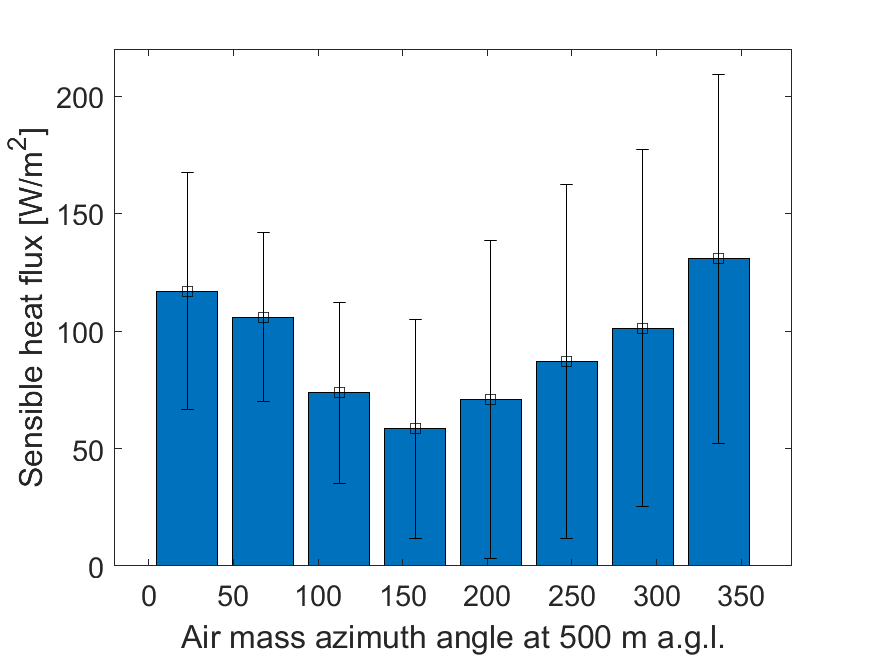
**

Fig. S5 Mean surface sensible heat flux [W/m^2^] from sonic anemometer at Warsaw site as a function of air mass azimuth angle (HYSPLIT) transported at 500 m a.g.l.

Table S1. Comparison of mean (05.2022-04.2025) aerosol scattering coefficient (ASC) [Mm^-1^] at 525, scattering Ångstrom exponent (SAE) at 635/450 nm, equivalent of black carbon concentration (eBC) at 520 nm [ng/m^3^], aerosol absorbing coefficient (AAC) [Mm^-1^] at 520 nm, and single-scattering albedo (SSA) at 525 nm. Values in the parentheses show weekend means.

|  | **Season** | **ASC [Mm^-1^]**  **525 nm** | **SAE**  **450/635 nm** | **eBC [ng/m^3^]** | **AAC**  **[Mm^-1^]** | **AAE** | **SSA** |
| --- | --- | --- | --- | --- | --- | --- | --- |
| Warsaw | winter | 84.1 (73.1) | 1.21 (1.17) | 1528 (1211) | 13.6 (10.9) | 1.6 (1.7) | 0.85 (0.86) |
|  | spring | 54.0 (51.2) | 1.49 (1.48) | 970 (866) | 8.3 (7.6) | 1.5 (1.6) | 0.86 (0.86) |
|  | summer | 35.6 (37.7) | 1.67 (1.76) | 819 (747) | 6.4 (6.0) | 1.4 (1.4) | 0.84 (0.85) |
|  | autumn | 61.7 (60.4) | 1.38 (1.39) | 1398 (1296) | 11.9 (11.2) | 1.5 (1.6) | 0.82 (0.83) |
|  | annual | 59.2 (56.1) | 1.43 (1.44) | 1182 (1034) | 10.1 (9.0) | 1.5 (1.6) | 0.84 (0.85) |
| Strzyżów | winter | 50.5 (48.5) | 1.39 (1.40) | 773 (696) | 6.5 (5.9) | 1.9 (1.9) | 0.88 (0.88) |
|  | spring | 35.9 (36.4) | 1.58 (1.57) | 526 (524) | 4.3 (4.3) | 1.7 (1.7) | 0.89 (0.89) |
|  | summer | 33.3 (34.0) | 1.72 (1.75) | 445 (435) | 3.2 (3.2) | 1.4 (1.5) | 0.91 (0.91) |
|  | autumn | 37.1 (36.7) | 1.39 (1.36) | 609 (585) | 4.9 (4.7) | 1.7 (1.7) | 0.88 (0.88) |
|  | annual | 39.3 (39.0) | 1.52 (1.52) | 590 (562) | 4.7 (4.5) | 1.7 (1.7) | 0.89 (0.89) |

Table S2. Comparison of mean (2018-2025) aerosol optical depth (AOD) at 500 nm and Ångstrom exponent (AE) at 440/870 nm for Warsaw and Strzyżow.

| **Season** | **Warsaw** | | **Strzyżów** | |
| --- | --- | --- | --- | --- |
|  | AOD 500 nm | AE 440/870 nm | AOD 500 nm | AE 440/870 nm |
| winter | 0.147 | 1.200 | 0.106 | 1.299 |
| spring | 0.182 | 1.279 | 0.148 | 1.346 |
| summer | 0.209 | 1.479 | 0.192 | 1.513 |
| autumn | 0.154 | 1.311 | 0.126 | 1.330 |
| annual | 0.185 | 1.361 | 0.159 | 1.407 |

Table S3. Pearson correlations coefficient, p-values, rmse, bias, and mean values of scattering Angstrom exponent for Warsaw and Strzyżów sites for inflow from four geographic directions during night (18-06 UTC) and day (06-18 UTC).

| AE | Time | r | p-Value | rmse | bias | Warsaw | Strzyżów |
| --- | --- | --- | --- | --- | --- | --- | --- |
| All data | day | 0.27 | 0.02 | 0.53 | -0.09 | 1.43 | 1.52 |
|  | night | 0.41 | 0.00 | 0.43 | -0.10 | 1.42 | 1.52 |
| West | day | 0.35 | 0.00 | 0.47 | -0.16 | 1.38 | 1.54 |
|  | night | 0.20 | 0.00 | 0.59 | -0.14 | 1.40 | 1.54 |
| East | day | 0.45 | 0.00 | 0.29 | 0.06 | 1.60 | 1.54 |
|  | night | 0.30 | 0.00 | 0.34 | 0.05 | 1.60 | 1.55 |
| North | day | 0.30 | 0.00 | 0.52 | -0.16 | 1.31 | 1.47 |
|  | night | 0.19 | 0.00 | 0.62 | -0.13 | 1.34 | 1.47 |
| South | day | 0.68 | 0.00 | 0.31 | -0.01 | 1.47 | 1.48 |
|  | night | 0.54 | 0.00 | 0.38 | -0.09 | 1.45 | 1.54 |

Table S4 Annual mean PBLH [m] from ERA5 at 00 and 12 UTC and rmse and mean bias of PBLH between Warsaw and Strzyżów site. In addition,, data are selected for 4 circulation directions.

|  | Warsaw | | Strzyżów | | rmse | | bias | |
| --- | --- | --- | --- | --- | --- | --- | --- | --- |
|  | 00 UTC | 12 UTC | 00 UTC | 12 UTC | 00 UTC | 12 UTC | 00 UTC | 12 UTC |
| all data | 311 | 1133 | 288 | 1043 | 209 | 419 | 23 | 90 |
| west | 348 | 1151 | 284 | 1038 | 222 | 429 | 64 | 113 |
| east | 248 | 1297 | 219 | 1125 | 172 | 453 | 29 | 172 |
| north | 368 | 1302 | 365 | 1197 | 224 | 380 | 3 | 105 |
| south | 234 | 821 | 267 | 851 | 177 | 367 | -33 | -30 |
